# Inhibition of PI3Kδ Enhances Poly I:C-Induced Antiviral Responses and Inhibits Replication of Human Metapneumovirus in Murine Lungs and Human Bronchial Epithelial Cells

**DOI:** 10.3389/fimmu.2020.00432

**Published:** 2020-03-11

**Authors:** Akitaka Fujita, Keiko Kan-o, Ken Tonai, Norio Yamamoto, Tomohiro Ogawa, Satoru Fukuyama, Yoichi Nakanishi, Koichiro Matsumoto

**Affiliations:** ^1^Research Institute for Diseases of the Chest, Graduate School of Medical Sciences, Kyushu University, Fukuoka, Japan; ^2^Department of Endoscopic Diagnostics and Therapeutics, Kyushu University Hospital, Fukuoka, Japan; ^3^Division of Intensive Care, Department of Anesthesiology and Intensive Care Medicine, Jichi Medical University School of Medicine, Tochigi, Japan

**Keywords:** poly I:C, phosphoinositide 3-kinase δ, IC87114, programmed death 1 ligand 1, interferon, bronchial epithelial cells, human metapneumovirus

## Abstract

Viral infections of the airway can exacerbate respiratory diseases, such as asthma or chronic obstructive pulmonary disease (COPD), and accelerate disease progression. Phosphoinositide 3-kinase (PI3K)δ, a class 1A PI3K, has been studied as a potential target for achieving anti-oncogenic and anti-inflammatory effects. However, the role of PI3Kδ in antiviral responses is poorly understood. Using a synthetic double-stranded RNA poly I:C and a selective PI3Kδ inhibitor IC87114, we investigated the role of PI3Kδ signaling in poly I:C-induced expression of the T lymphocyte-inhibitory molecule programmed death 1 ligand 1 (PD-L1), inflammatory responses and antiviral interferon (IFN) responses. C57BL/6N mice were treated with IC87114 or vehicle by intratracheal (i.t.) instillation followed by i.t. administration of poly I:C. Poly I:C increased PD-L1 expression on epithelial cells, lymphocytes, macrophages, and neutrophils in the lungs and IC87114 suppressed poly I:C-induced PD-L1 expression on epithelial cells and neutrophils possibly via inhibition of the Akt/mTOR signaling pathway. IC87114 also attenuated poly I:C-induced increases in numbers of total cells, macrophages, neutrophils and lymphocytes, as well as levels of KC, IL-6 and MIP-1β in bronchoalveolar lavage fluid. Gene expression of IFNβ, IFNλ_2_ and IFN-stimulated genes (ISGs) were upregulated in response to poly I:C and a further increase in gene expression was observed following IC87114 treatment. In addition, IC87114 enhanced poly I:C-induced phosphorylation of IRF3. We assessed the effects of IC87114 on human primary bronchial epithelial cells (PBECs). IC87114 decreased poly I:C-induced PD-L1 expression on PBECs and secretion of IL-6 and IL-8 into culture supernatants. IC87114 further enhanced poly I:C- induced increases in the concentrations of IFNβ and IFNλ_1/3_ in culture supernatants as well as upregulated gene expression of ISGs in PBECs. Similar results were obtained in PBECs transfected with siRNA targeting the PIK3CD gene encoding PI3K p110δ, and stimulated with poly I:C. In human metapneumovirus (hMPV) infection of PBECs, IC87114 suppressed hMPV-induced PD-L1 expression and reduced viral replication without changing the production levels of IFNβ and IFNλ_1/3_ in culture supernatants. These data suggest that IC87114 may promote virus elimination and clearance through PD-L1 downregulation and enhanced antiviral IFN responses, preventing prolonged lung inflammation, which exacerbates asthma and COPD.

## Introduction

Asthma and chronic obstructive pulmonary disease (COPD) are chronic respiratory diseases that are major causes of morbidity and mortality worldwide (1, 2). Airway infection by viruses including rhinovirus, respiratory syncytial virus, human metapneumovirus, and influenza virus is one of the leading causes of asthma and COPD exacerbation, which can lead to heightened inflammation and a general decline in health status (3, 4). These viruses possess single-stranded RNA genomes and generate double-stranded RNA (dsRNA) in host cells during replication (excluding influenza virus which has a unique 5′-triphosphate dsRNA panhandle structure). These dsRNAs, known as pathogen-associated molecular patterns, bind to Toll-like receptor (TLR) 3 in endosomes of antigen-presenting cells including bronchial epithelial cells, as well as to cytosolic RNA helicase retinoic acid-inducible gene-I or melanoma differentiation-associated gene 5 (MDA5) to subsequently activate antiviral immune responses (5–7). A synthetic dsRNA analog, polyinosinic-polycytidylic acid (poly I:C), is recognized by TLR3 and MDA5 and subsequent signaling leads to activation of transcription factors including interferon regulatory factor-3 (IRF3) and nuclear factor kappa-light-chain-enhancer of activated B cells (NF-κB), resulting in secretion of type I and III interferons (IFNs) and proinflammatory cytokines and chemokines, respectively (8–11). Type I IFNs stimulate neighboring cells to express IFN-regulated genes (IRGs), inducing an antiviral state (12).

Adaptive antiviral immunity is initiated by interactions between antigen-presenting cells and T cells. These interactions include binding of an array of co-stimulatory or co-inhibitory molecules including B7 family members and their putative receptors (13). Programmed cell death 1 ligand 1 (PD-L1) is a B7-family co-inhibitory molecule, which is constitutively expressed on antigen presenting cells. Ligation of programmed cell death 1 (PD-1) by PD-L1 on activated T cells causes T cell impairment and exhaustion (14, 15). Some investigators reported that inhibition of the PD-1/PD-L1 pathway restored virus-specific CD8^+^ T cell activity and reduced viral loads in murine models of acute or chronic viral infection, suggesting that PD-L1 upregulation on infected cells is responsible for CD8^+^ T cell exhaustion and failure to achieve viral clearance (16–19). We have previously shown that poly I:C stimulation enhanced the expression of PD-L1 on human and murine bronchial epithelial cells; these cells are targeted by respiratory viruses for their replication (20–22). Given the high expression of PD-1 on virus-specific CD8^+^ T cells, dsRNA-induced PD-L1 expression on infected cells may prevent CD8^+^ T cells from eliminating these cells and thereby promote the spread of virus to neighboring cells.

Phosphoinositide 3-kinases (PI3Ks) can be divided into three distinct classes (I, II, and III) according to their structures and substrate specificities. PI3Ks affect important signaling pathways via several downstream molecules such as Akt, 3′-phosphoinositide dependent kinase 1, the mechanistic target of rapamycin (mTOR), and glycogen synthase kinase 3 which control biological functions including cell proliferation, differentiation, migration, and metabolism (23, 24). There are three isoforms of class IA PI3Ks in mammalian cells: α, β, and δ. Each class IA PI3K is composed of a catalytic subunit (p110α, p110β, or p110δ) and a SH2 domain-containing regulatory subunit (p85α, p55α, p50α, p85β, and p55γ) (25). The catalytic subunits p110α and p110β are broadly expressed, while p110δ is predominantly expressed in cells of the myeloid and lymphoid lineages. Recently, a primary immunodeficiency caused by mutations in the *PIK3CD* or *PIK3R1* genes (encoding p110δ and p85α, respectively) was reported and called activated PI3K delta syndrome (APDS) (26–28). The *PIK3CD* or *PIK3R1* mutations increased PI3Kδ activities and patients with APDS suffered from infectious complications such as recurrent bacterial respiratory infections and severe or persistent infections by herpesviruses, including Epstein-Barr virus, cytomegalovirus, and varicella-zoster virus (29). Although APDS phenotypes suggest that high PI3Kδ activities aggravate viral infection, the roles of PI3Kδ signaling in modulating co-inhibitory molecule (e.g., PD-L1) expression and antiviral IFN responses are poorly understood.

Human metapneumovirus (hMPV) discovered in 2001 has been identified as the secondary cause of acute lower respiratory infection in children, and moreover recently implicated in exacerbations of asthma and COPD (30, 31). There is currently no licensed vaccine or therapy available for hMPV. Previous several mouse studies have shown that hMPV is able to persist in respiratory infected cells by inhibiting innate immune responses and causing CD8^+^ T cells impairment mediated by PD-1 (32, 33). While animal and human *in vivo* studies confirmed that airway epithelium can sustain hMPV infection and replication (34, 35), there have been limited studies describing hMPV infection of primary culture bronchial epithelial cells.

In this study, we evaluated the effects of the PI3Kδ inhibitor IC87114 on PD-L1 expression, inflammatory responses, and antiviral IFN responses in lung cells following intratracheal (i.t.) administration of poly I:C in mice. We also assessed the effects of IC87114 on human primary bronchial epithelial cells (PBECs) stimulated by poly I:C or infected with hMPV.

## Materials and Methods

### Animals

Male 8-week-old C57BL/6N mice were purchased from Japan SLC, Inc. (Shizuoka, Japan). Mice were housed under specific pathogen-free conditions. The study protocol was approved by the Kyushu University Animal Care and Use Committee (A19-021-1).

### Intratracheal Administration of Poly I:C

Mice were anesthetized intraperitoneally with 80 mg/kg ketamine and 16 mg/kg xylazine and their tracheas were cannulated using 22-gauge catheters. IC87114 (1 mg/kg body weight in 25 μL total volume; BioVision, Milpitas, CA) or vehicle was administered by i.t. instillation followed by i.t. administration of poly I:C (3 μg in 30 μL total volume; Innaxon, Tewkesbury, United Kingdom) or vehicle. Mice were sacrificed 24 h following i.t. administration and lungs were removed following bronchoalveolar lavage. IC87114 was diluted to 1 mg/mL with 0.1% dimethyl sulfoxide. Poly I:C was diluted with 0.9% NaCl. Sham-treated mice were used as controls.

### Flow Cytometry

Lungs were minced and single-cell suspensions were prepared. Cells were suspended in 100 μL of phosphate-buffered saline (PBS) containing 0.5% bovine serum albumin (BSA) and preincubated with anti-mouse CD16/CD32 antibody (BD Biosciences, San Jose, CA) for 15 min to prevent non-specific binding via the Fcγ receptor. The cells were washed and suspended in 100 μL of PBS containing 0.5% BSA and the following antibodies for 30 min: phycoerythrin (PE) anti-mouse CD326 (epithelial cell adhesion molecule; Ep-CAM) (BioLegend, San Diego, CA), peridinin-chlorophyll-protein (PerCP)/Cy5.5 anti-mouse CD45 (BioLegend), fluorescein isothiocyanate (FITC) anti-mouse CD11b (eBioscience, San Diego, CA), FITC anti-mouse CD11c (eBioscience), PE/Cy7 anti-mouse CD274 (PD-L1) (eBioscience), and allophycocyanin (APC)/Cy7 anti-mouse CD279 (PD-1) (BioLegend). Isotype controls were included as appropriate to facilitate gating of each population. Living cells were analyzed by addition of propidium iodide (PI) to samples in the first experiment and via the forward scatter (FSC) threshold in subsequent experiments. Cells were thoroughly washed and analyzed using a BD FACSVerse flow cytometer with FACSuite software (Becton Dickinson, Franklin Lakes, NJ). One hundred thousand events were acquired in list mode. Epithelial cells, lymphocytes, macrophages, and neutrophils were identified as CD11b/CD11c^low^ CD45^low^ CD326 (Ep-CAM)^high^, CD45^high^ CD11b/CD11c^low^, CD45^high^ CD11b/CD11c^high^ side scatter (SSC)-A^low^, and CD45^high^ CD11b/CD11c^high^ SSC-A^high^, respectively. In some experiments, cells were suspended in PBS containing 0.5% BSA with both APC anti-mouse CD3 (BioLegend) and PE anti-mouse CD19 (BioLegend) antibodies, or both APC anti-mouse Ly-6C (BD Biosciences) and PE anti-mouse Ly-6G (BioLegend) antibodies instead of PE anti-mouse CD326, PE/Cy7 anti-mouse CD274, and APC/Cy7 anti-mouse CD279 antibodies in order to validate gating strategy for lymphocytes, neutrophils and macrophages. In some experiments, cells were sorted using a BD FACSAria SORP (Becton Dickinson) and centrifuged at 72× g for 5 min. Pelleted cells were stained with Diff-Quick and observed using an optical microscope (Nikon Eclipse Ni-U, Nikon Corporation, Tokyo, Japan).

For *in vitro* experiments, PBECs or BEAS-2B were cultured to semi-confluence in 12-well plates. Cells were incubated in 100 μL of PBS containing 0.5% BSA and PE anti-human PD-L1 (clone: MIH1; Invitrogen, Carlsbad, CA) at room temperature for 30 min. The cells were thoroughly washed and analyzed using a BD FACSVerse flow cytometer with FACSuite software (Becton Dickinson). Ten thousand events were acquired in list mode with debris excluded by the FSC threshold.

For the analysis of cell viability, resuspended lung cells or PBECs were incubated with PI and non-viable cells stained with PI were counted in a flow cytometer with debris excluded by the FSC threshold.

### Culture and Treatment of Primary Bronchial Epithelial Cells (PBECs) and BEAS-2B

PBECs were obtained during routine fibreoptic bronchoscopy from never-smoker patients with normal lung function and pulmonary nodules. Bronchial brushings were performed from healthy lobes without a pulmonary nodule. All bronchial brushings were obtained from the same anatomical region (bronchial generations 4–7). The study protocol was approved by the Kyushu University Institutional Review Board for Clinical Research (29-170). All subjects provided written informed consent in accordance with the principles laid out in the Declaration of Helsinki.

Cells were cultured at 37°C/5% CO_2_ under submerged conditions on flasks coated with collagen (Cell Applications, Inc. San Diego, CA) in supplemented bronchial epithelial growth medium (BEGM; Lonza, Basel, Swiss) and used within four passages. PBECs (~80% confluency) were pretreated with 10 μM IC87114 or vehicle for 1 h, then stimulated by addition of 1 μg/mL poly I:C to the culture medium. In virus infection experiments, 10 μM IC87114 was added to cells for 1 h prior to hMPV infection, and then following infection. PBECs were cultured in BEGM without hydrocortisone for at least 24 h prior to stimulation or infection.

In some experiments, human bronchial epithelial cells, BEAS-2B, were cultured in DMEM/F12 (gibco, Thermo Fisher Scientific, San Diego, CA) with 10% FBS and 1% penicillin-streptomycin and incubated at 37°C/5% CO_2_. When cells reached semi-confluence, they were pretreated with 10 or 100 nM rapamycin (LC Laboratories, Woburn, MA) or vehicle for 1 h, then stimulated by addition of 3 μg/mL poly I:C to the culture medium.

### Gene Expression in Mouse Lungs and Human Bronchial Epithelial Cells

Total RNA was isolated from mouse lungs, PBECs or BEAS-2B using TRIzol® Reagent (Thermo Fisher Scientific) or TRI Reagent® (Molecular Research Center, Inc, Cincinnati, OH). Reverse transcription was performed using Multiscribe Reverse Transcriptase (Invitrogen). Real-time quantitative reverse-transcriptase PCR analyses were performed using SYBR Premix Ex Taq II (Takara, Shiga, Japan) and a Thermal Cycler Dice Real Time System II (Takara). Target gene expression levels were normalized to expression of glyceraldehyde-3-phosphate dehydrogenase (GAPDH) or 18S rRNA. Primer sequences are provided in Supplementary Table 1.

### Western Blotting

Proteins were extracted from mouse lungs using TRIzol® Reagent (Thermo Fisher Scientific) according to the manufacturer's instructions. For *in vitro* experiments, PBECs cultured to semi-confluence in 6-well plates were lysed using Pierce® RIPA Buffer (Thermo Fisher Scientific) according to the manufacturer's instructions. Ten micrograms of the protein sample were denatured, separated by SDS-PAGE, and transferred to polyvinylidene difluoride membranes. Membranes were blocked with TBS-Tween (10 mM Tris, 150 mM NaCl, 0.05% Tween 20, pH 8.0) containing 5% skimmed milk for 1 h at room temperature. After blocking, blotting was performed with anti-IκBα (Cell signaling Technology, Beverly, MA), anti-phospho-IκBα (Ser32, Cell signaling Technology), anti-v-akt murine thymoma viral oncogene homolog (Akt) (Cell signaling Technology), anti-phaspho-Akt (Ser473, Cell signaling Technology), anti-IRF3 (Cell signaling Technology), anti-phospho-IRF3 (Ser379, Cell signaling Technology), anti-PI3 Kinase p110δ (Cell Signaling Technology), or anti-β-actin (Santa Cruz Biotechnology, Dallas, TX) antibodies at 4°C overnight. Membranes were washed three times with TBS-Tween and then incubated with a horseradish peroxidase-conjugated secondary antibody for 60 min at room temperature. Membranes were washed three times with TBS-Tween, and specific bands were visualized using ImmunoStar® LD (Wako, Osaka, Japan) according to the manufacturer's instructions. All blots were imaged using the ChemiDoc™ XRS+ system (Bio-Rad Laboratories, Inc. Hercules, CA). Densitometric analysis of band intensities was performed using Image J.

### Collection of Bronchoalveolar Lavage Fluid (BALF)

Mice were given lethal doses of pentobarbital and their lungs were gently lavaged once with 1 mL of 0.9% saline via the tracheal cannula. Total and differential cell counts in BALF were performed as described previously (36).

### Detection of Cytokines and Chemokines

BALF was centrifuged at 250× g for 10 min, and the supernatants were stored at −80°C. Levels of mouse keratinocyte-derived chemokine (KC; detection limit 15.6–1,000 pg/mL), interleukin (IL)-6 (detection limit 7.8–500 pg/mL) and macrophage inflammatory protein-1β (MIP-1β; detection limit 7.8–500 pg/mL) in BALF were measured by enzyme-linked immunosorbent assay (ELISA) (R&D systems, Inc. Minneapolis, MN). Levels of human IL-6 (detection limit 9.38–600 pg/mL), IL-8 (detection limit 31.2–2,000 pg/mL), IFN-β (detection limit 7.81–500 pg/mL), and IFN-λ_1/3_ (detection limit 62.5–4,000 pg/mL) in cell culture supernatants were determined using ELISA (R&D systems, Inc.) Cell supernatants were diluted as appropriate for measurement.

### Preparation and Transfection of Small Interfering RNA (siRNA)

PBECs cultured in 6-well plates (60–80% confluence) were transiently transfected with 10 nM PIK3CD siRNA (Silencer® Select Validated siRNA, s10529; Ambion, Life Technologies, Carlsbad, CA) or 10 nM negative control (NC) siRNA (Silencer® Select Negative Control siRNA, 4390843; Ambion, Life Technologies) using Lipofectamine® RNAiMAX Reagent (Invitrogen) according to the manufacturer's instructions. Lipofectamine® RNAiMAX Reagent or siRNA were diluted in BEGM without hydrocortisone and antibiotics. Cells were used for experiments at 48 h after transfection.

### Virus Infection of Cultured Cells

The CAN97-83 strain of hMPV was used in most experiments, propagated in Vero E6 cells (CRL-1586; ATCC, Manassas, VA) and virus titer was determined by TCID_50_ assay. In brief, virus was prepared in serum free MEM (gibco) containing 5 μg/ml trypsin (gibco) by infecting cells in a 10-cm culture dish at a multiplicity of infection (MOI) of 0.05. After absorption for 2 h at 37°C/5%CO_2_, serum free MEM containing 5 μg/ml trypsin was added. Serum free medium containing 5 μg/ml trypsin was changed every other day during incubation and cells were incubated until 70–90% cytopathic effect was apparent, usually withhin 7 days. Cells and culture supernatant were collected and virus was obtained by freeze-thawing the cells. This strain of hMPV has been used in other studies (31) and we did not attempt further purification since this may change key properties of the virus (37). Virus was quantified by immunostaining assay. Briefly, semi-confluent Vero E6 monolayers in a 24 well plate were infected with 200 μl of 10-fold serial dilutions. Five μg/ml trypsin was added in serum free MEM during infection. After absorption for 2 h at 37°C/5%CO_2_, 200 μl of MEM containing 5% FBS was added and then cells were incubated. After 6 days, infected wells were identified by using monse anti-hMPV monoclonal antibody (MAB8510; EMD Millipore, Temecula, CA) followed by a horseradish peroxidase-labeled goat anti-mouse secondary antibody (abcam, Cambridge, UK). Cells were visualized using DAB peroxidase substrate (Vector Laboratories, Inc. Burlingame, CA) and the TCID_50_/ml was determined using standard methods. The CAN97-83 strain of recombinant hMPV with green fluorescent protein (hMPV-GFP) was purchased from ViraTree (http://www.viratree.com). Control experiments using UV-inactivated hMPV or hMPV-GFP were included to confirm that responses were a result of hMPV infection and not from factors remaining in the culture media following hMPV propogation. To UV-irradiate virus, hMPV was exposed to a short-wavelength (254 nm) UV lamp at a distance of 5 cm for 15 min.

PBECs (~80% confluency) were washed with PBS and infected with hMPV or hMPV-GFP in BEGM without hydrocortisone for 1 h at a MOI of 0.1. Cell monolayers were washed, then incubated in BEGM without hydrocortisone for 24–72 h. Cells infected with hMPV-GFP were observed using fluorescence microscopy (BZ-X800, KEYENCE, Osaka, Japan). The number of cells infected with hMPV-GFP were counted in four random microscopic fields (4× objective lens) per well and the mean value was calculated.

Standard biosecurity and institutional safety procedures were adhered to during this study.

### Statistical Analyses

Unless otherwise stated, data were expressed as means ± standard deviations (SDs). The Mann-Whitney U-test was used for comparisons between two groups. Comparisons of three or more groups were conducted using one-way analysis of variance (ANOVA) or two-way ANOVA followed by Tukey's multiple comparisons test. Correlations were examined using Spearman correlation. All statistical analyses were conducted using GraphPad Prism 8 software (GraphPad Software, San Francisco, CA). Differences were considered statistically significant at *p* < 0.05.

## Results

### Poly I:C Upregulates PD-L1 Expression on Lung Cells in Mice

We have previously showed that PD-L1 on the epithelium in mouse lung was significantly upregulated 24 and 72 h following i.t. poly I:C administration, while numbers of neutrophils in the BALF increased 24 h and then diminished promptly 48 h following i.t. poly I:C administration (22). Therefore, we evaluated PD-L1 expression and enumerated cells in BALF 24 h following poly I:C administration.

**Figure 1:**
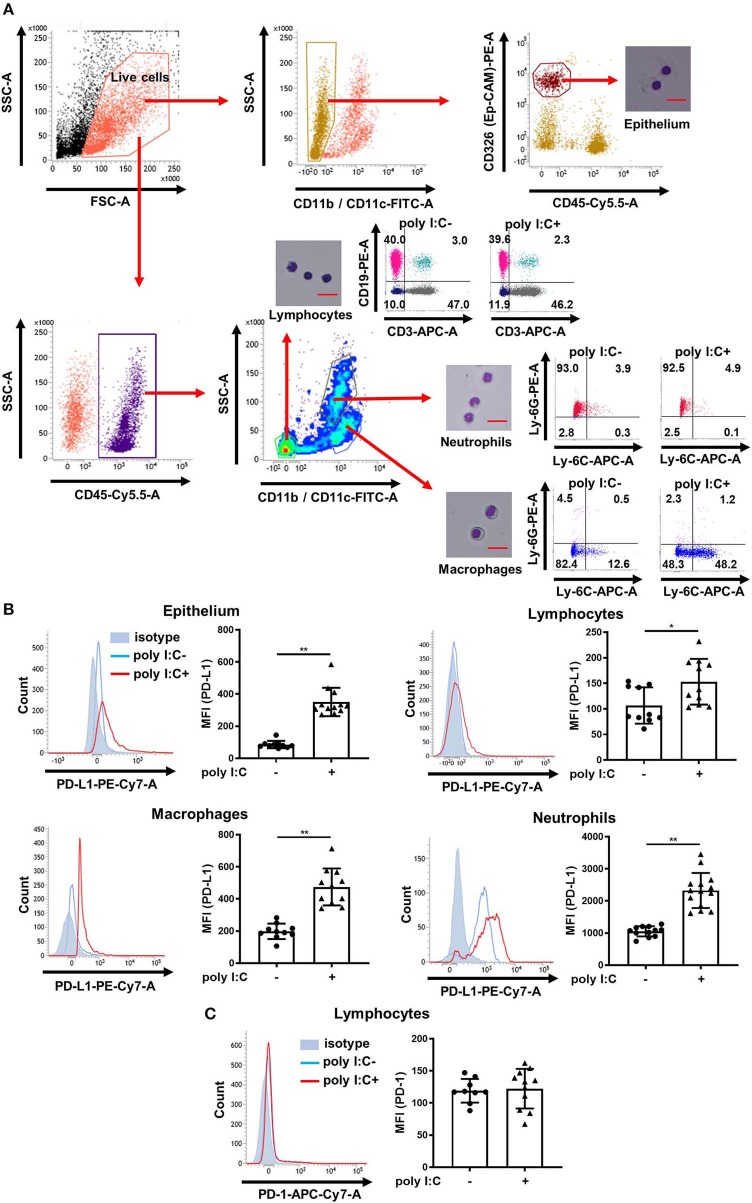
Poly I:C-induced upregulation of PD-L1 on mouse lung cells. **(A)** Flow cytometry gating strategy to distinguish epithelial cells, lymphocytes, macrophages, and neutrophils in mouse lungs. Sorted and pelleted cells were stained with Diff-Quick and observed using an optical microscope. Scale bar, 20 μm. **(B,C)** Poly I:C or vehicle was administered intratracheally to mice and PD-L1 **(B)** or PD-1 **(C)** expression on lung cells was analyzed 24 h following administration using flow cytometry. Representative histograms are shown. SSC, side scatter; FSC, forward scatter; MFI, mean fluorescence intensity. All results are representative of at least three independent experiments. Data represent means ± SDs (*n* = 6–14 per group). *p < 0.01, **p < 0.001 by the Mann–Whitney *U*-test.

We measured PD-L1 and PD-1 expression on epithelium, lymphocytes, macrophages, and neutrophils in the lungs of poly I:C-treated and untreated mice using the flow cytometry gating strategy outline in Figure 1A. To validate our method, lung cells were sorted and examined by microscopy, or further classified using specific antibodies (Figure 1A). Approximately 90% of identified lymphocytes were positive for only CD3 or CD19 and more than 90% of identified neutrophils were positive for Ly-6G. More than 90% of identified macrophages were negative for Ly-6G and the Ly-6G ^low^/Ly-6C ^high^ population (inflammatory macrophages) was increased in poly I:C-treated group (Figure 1A). Under unstimulated conditions, PD-L1 was consistently expressed on epithelium, macrophages, and neutrophils, but not on lymphocytes (Figure 1B, histograms). Administration of poly I:C i.t. significantly increased the expression of PD-L1 on lung cells at 24 h post-stimulation (Figure 1B). By contrast, PD-1 was consistently expressed on lymphocytes and poly I:C stimulation had no effect on its expression (Figure 1C).

### A PI3Kδ Inhibitor Attenuates Poly I:C-Induced PD-L1 Expression on Epithelium and Neutrophils in Mouse Lungs

**Figure 2:**
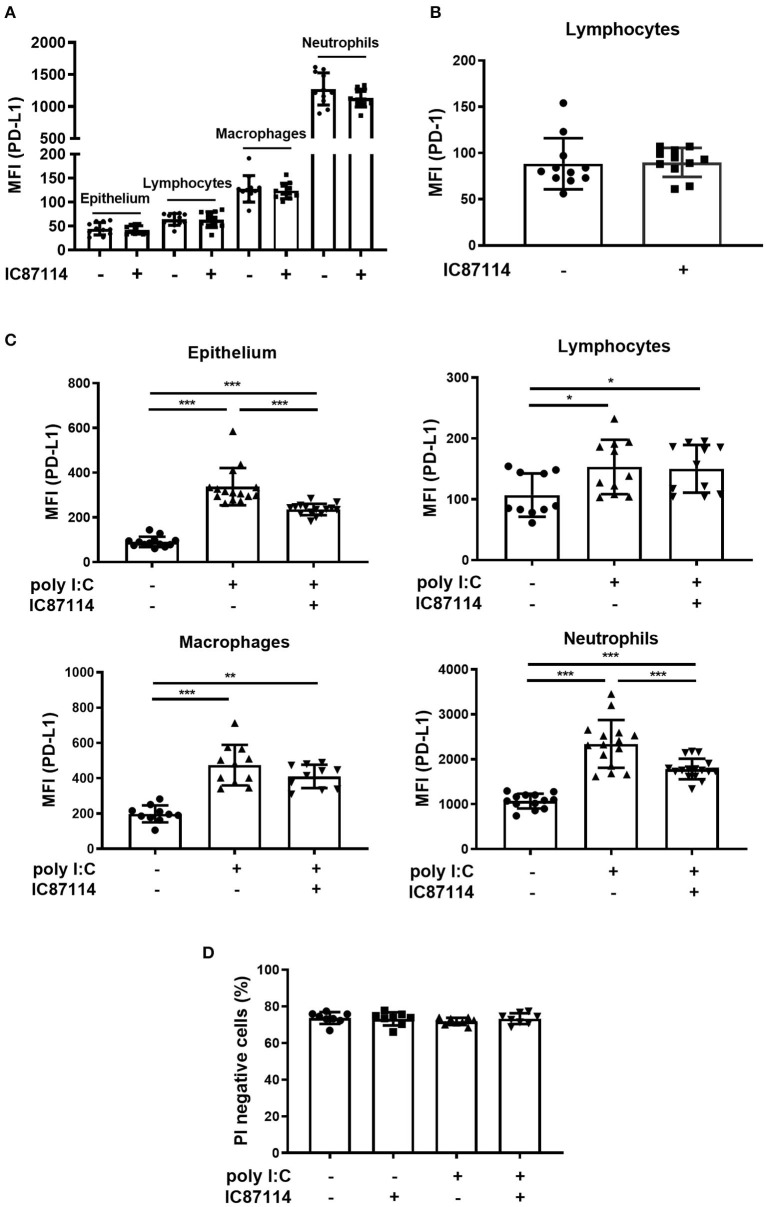
A PI3Kδ inhibitor attenuated poly I:C-induced upregulation of PD-L1 on mouse lung cells. **(A,B)** IC87114 was administered intratracheally (i.t.) to mice and PD-L1 **(A)** or PD-1 **(B)** expression on mouse lung cells was analyzed 24 h following treatment using flow cytometry. **(C,D)** IC87114 or vehicle was administered i.t. to mice followed by i.t. administration of poly I:C or vehicle. PD-L1 expression **(C)** on lung cells or cell viability **(D)** was analyzed 24 h following administration using flow cytometry. MFI, mean fluorescence intensity. All results are representative of at least three independent experiments. Data represent means ± SDs (*n* = 8–16 per group). *p < 0.05, **p < 0.01, ***p < 0.001 by the Mann–Whitney U-test or one-way ANOVA as appropriate.

We previously reported that poly I:C increased the expression of PD-L1 on BEAS-2B human bronchial epithelial cells and that the selective PI3Kδ inhibitor IC87114 partially suppressed poly I:C-induced PD-L1 expression (21, 38). Therefore, we investigated whether IC87114 attenuated poly I:C-induced PD-L1 expression *in vivo*. Treatment with IC87114 alone did not affect PD-L1 expression on mouse lung cells or PD-1 expression on lymphocytes (Figures 2A,B). Twenty-four hours following poly I:C administration, PD-L1 expression was significantly upregulated on epithelium, lymphocytes, macrophages, and neutrophils in mouse lungs (Figure 2C). Treatment with IC87114 significantly attenuated poly I:C-induced upregulation of PD-L1 on epithelium and neutrophils, but not its upregulation on lymphocytes and macrophages (Figure 2C). Cell viability was evaluated by PI exclusion assay 24 h following poly I:C administration. Treatment with IC87114 alone, poly I:C alone, or IC87114 plus poly I:C did not affect cell viability (Figure 2D and Supplementary Figure 1A).

### A PI3Kδ Inhibitor Suppresses Poly I:C-Induced PD-L1 Expression via Inhibition of the Akt/mTOR Signaling Pathway

**Figure 3:**
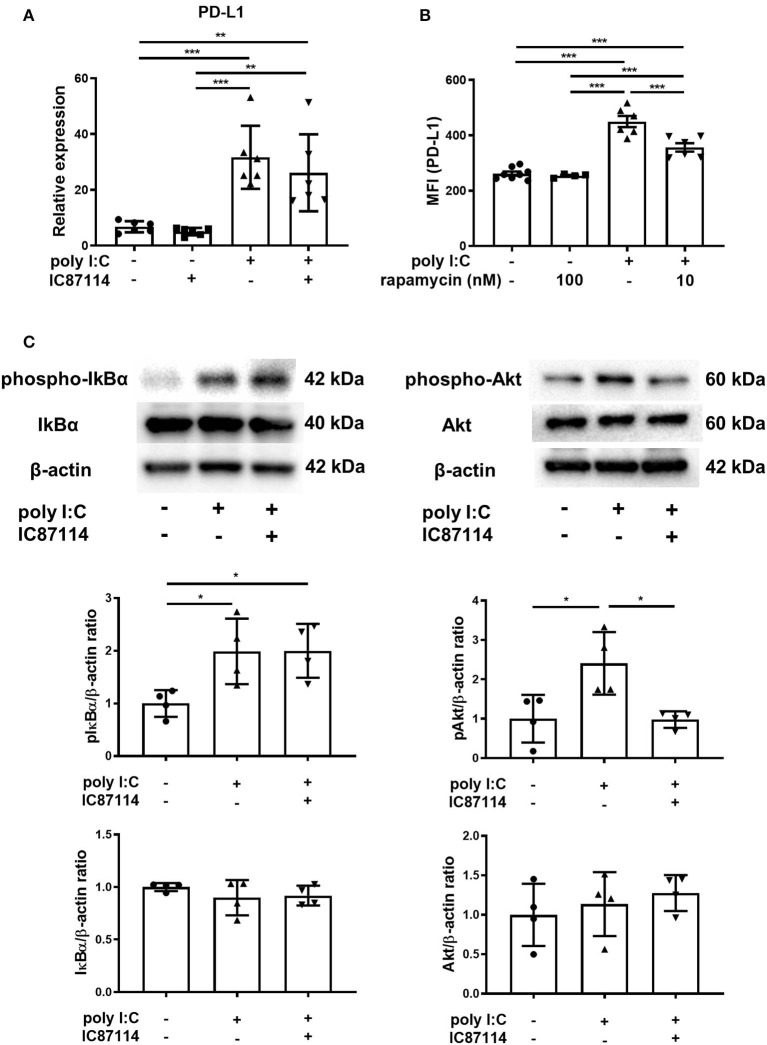
A PI3Kδ inhibitor suppressed poly I:C-induced PD-L1 expression via inhibition of Akt/mTOR signaling pathway. **(A)** PBECs were pretreated with IC87114 or vehicle for 1 h, then stimulated with poly I:C. Cell lysates for RNA extraction were collected 6 h following stimulation and real-time quantitative reverse-transcriptase PCR was performed. Target gene expression levels were normalized to those of 18S rRNA. **(B)** BEAS-2B cells were pretreated with rapamycin or vehicle for 1 h, then stimulated with poly I:C. PD-L1 expression was analyzed 24 h following administration using flow cytometry. MFI, mean fluorescence intensity. **(C)** IC87114 or vehicle was administered intratracheally (i.t.) to mice followed by i.t. administration of poly I:C or vehicle. Lungs were collected 1 h following administration. Phosphorylated IκBα, total IκBα, phosphorylated Akt and total Akt were detected by western blotting and quantitated using densitometry. All results are representative of at least two independent experiments. Data represent means ± SDs (*n* = 4–6 per group). *p < 0.05, **p < 0.01, ***p < 0.001 by one-way ANOVA.

In our previous studies using BEAS-2B, the selective PI3Kδ inhibitor IC87114 suppressed poly I:C-induced PD-L1 expression without affecting the phosphorylation of IκBα although the NF-κB pathway played an essential role in poly I:C-induced upregulation of PD-L1 (21, 38). Further experiments were conducted to explore the inhibitory mechanism of PD-L1 by IC87114 *in vitro* and *in vivo*. Poly I:C increased PD-L1 gene expression in human bronchial epithelial cells at 6 h following poly I:C stimulation and combined treatment with IC87114 and poly I:C did not alter the levels of *PD-L1* gene expression, suggesting that PI3Kδ provides translational control of PD-L1 (Figure 3A). Lastwika et al. reported that activation of the PI3K/Akt/mTOR pathway regulates translationally PD-L1 expression in bronchial epithelial cells (39). Similarly in our study, treatment with the mTOR inhibitor rapamycin suppressed poly I:C-induced PD-L1 on human bronchial epithelial cells 24 h following poly I:C stimulation (Figure 3B). *In vivo*, an hour following poly I:C administration, IκBα and Akt was phosphorylated in poly I:C-treated mouse lungs (Figure 3C). Treatment with IC87114 significantly suppressed Akt phosphorylation by poly I:C, but not IκBα phosphorylation (Figure 3C). These results indicate that PI3Kδ may contribute translational induction of PD-L1 expression via the Akt/mTOR pathway in poly I:C-stimulated bronchial epithelial cells.

### A PI3Kδ Inhibitor Suppresses Poly I:C-Induced Influx of Inflammatory Cells and Production of Pro-Inflammatory Chemokines and Cytokines in BALF

**Figure 4:**
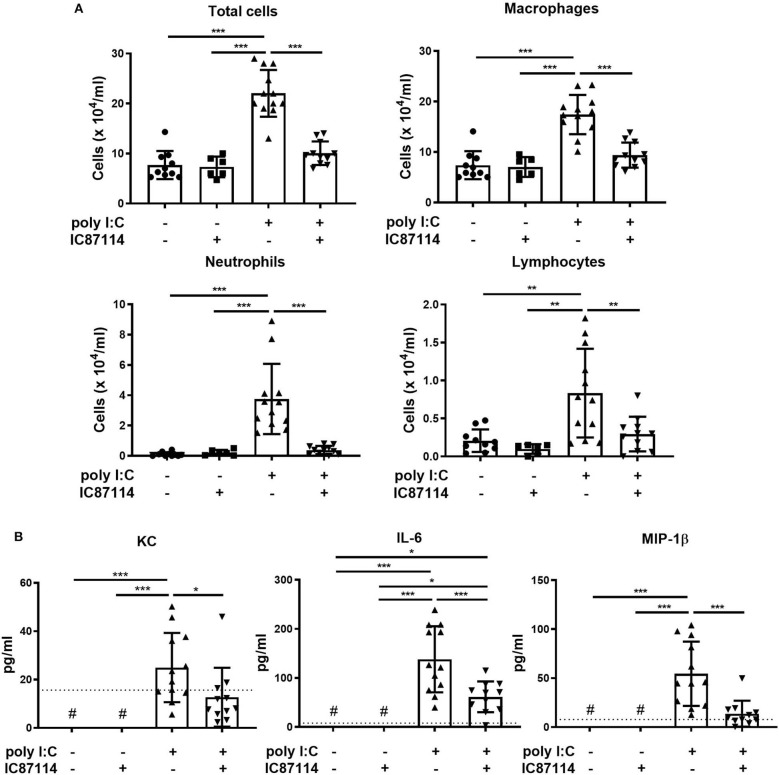
A PI3Kδ inhibitor suppressed poly I:C-induced influx of inflammatory cells and increases in pro-inflammatory chemokines and cytokines in BALF. IC87114 or vehicle was administered intratracheally (i.t.) to mice followed by i.t. administration of poly I:C or vehicle. BALF were collected 24 h following administration. **(A)** Cells were enumerated in BALF. **(B)** Pro-inflammatory cytokine and chemokine levels in BALF were measured by ELISA. The dotted line shows the lower limit of detection. ^#^Levels in all samples were below the detection limit. All results are representative of at least three independent experiments. Data represent means ± SDs (*n* = 6–12 per group). *p < 0.05, **p < 0.01, ***p < 0.001 by one-way ANOVA.

Next, we analyzed the effects of IC87114 on inflammatory cell infiltration and production of pro-inflammatory chemokines and cytokines in BALF. Total cell numbers in BALF were increased nearly 3-fold compared with controls 24 h following poly I:C administration. Treatment with IC87114 suppressed poly I:C-induced influx of inflammatory cells to the same levels as controls (Figure 4A). Poly I:C stimulation significantly increased the numbers of BALF macrophages, neutrophils and lymphocytes, but not the numbers of eosinophils (Figure 4A and data not shown). Poly I:C-induced increases in numbers of macrophages, neutrophils and lymphocytes were attenuated by IC87114 and treatment with IC87114 alone did not affect cell numbers in BALF (Figure 4A).

Levels of KC, IL-6, and MIP-1β in BALF were analyzed as representative pro-inflammatory chemokines and cytokines induced by TLR3 and MDA5 signaling. Poly I:C significantly increased the concentration of KC, IL-6 and MIP-1β in BALF 24 h following administration, and treatment with IC87114 attenuated poly I:C-induced increases in KC, IL-6 and MIP-1β (Figure 4B).

### A PI3Kδ Inhibitor Enhances Poly I:C-Induced IFN Responses by Promoting IRF3 Phosphorylation in Mouse Lungs

**Figure 5:**
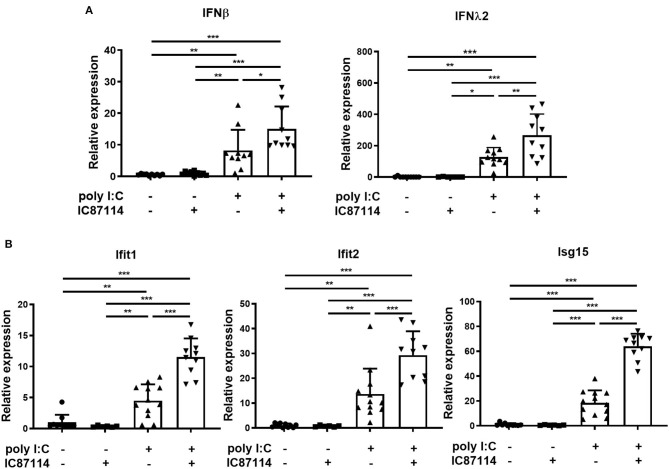
A PI3Kδ inhibitor enhanced poly I:C-induced IFN responses in mouse lung. IC87114 or vehicle was administered intratracheally (i.t.) to mice followed by i.t. administration of poly I:C or vehicle. Lungs were collected at 6 **(A)** or 24 h **(B)** post-administration and RNA was extracted. Real-time quantitative reverse-transcriptase PCR was performed and target gene expression levels were normalized to those of GAPDH. Data represent means ± SDs (*n* = 6–12 per group). All results are representative of at least three independent experiments. *p < 0.05, **p < 0.01, ***p < 0.001 by one-way ANOVA.

Types I and III IFNs play important roles in immune responses to viral infection (12, 40). Thus, we examined the effects of IC87114 on type I and type III IFNs responses in mouse lungs using real-time quantitative PCR. Six hours following poly I:C administration, expression of *IFN*β and *IFN*λ_2_ was significantly increased compared with controls and with mice treated with IC87114 alone (Figure 5A). Furthermore, combined treatment with IC87114 and poly I:C enhanced expression of *IFN*β and *IFN*λ_2_ to a greater degree than poly I:C treatment alone (Figure 5A). Expression of IRGs such as IFN-induced protein with tetratricopeptide repeats (*Ifit*)*1, Ifit2*, and IFN-stimulated gene (*ISG*)*15* were analyzed and similar results were obtained. Significant upregulation of *Ifit1, Ifit2*, and *ISG15* expression was observed 24 h following poly I:C administration. Combined treatment with IC87114 and poly I:C greatly enhanced the expression of these IRGs compared with poly I:C-treated mice (Figure 5B).

**Figure 6:**
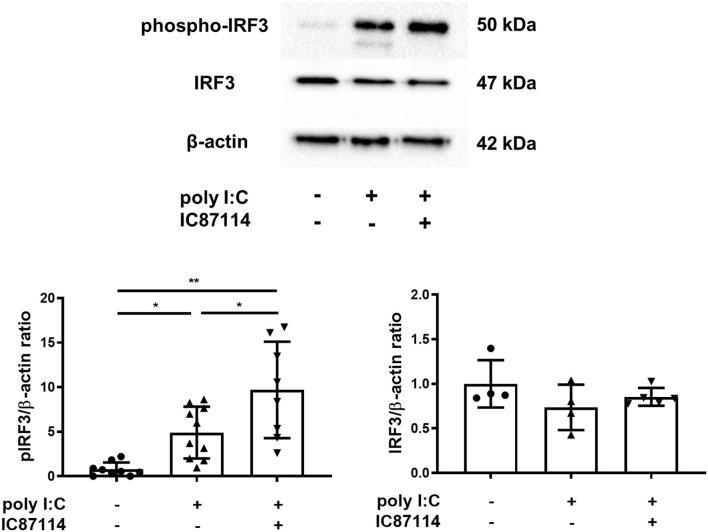
A PI3Kδ inhibitor enhanced poly I:C-induced phosphorylation of IRF3 in mouse lung. IC87114 or vehicle was administered intratracheally (i.t.) to mice followed by i.t. administration of poly I:C or vehicle. Lungs were collected 3 h following administration. Phosphorylated IRF3 and total IRF3 were detected by western blotting and quantitated using densitometry. All results are representative of at least two independent experiments. Data represent means ± SDs (*n* = 4–5 per group). *p < 0.05, **p < 0.001 by one-way ANOVA.

Previous studies showed that the production of early IFNs results from the phosphorylation and translocation of available IRF3 into the nucleus and does not require production of new IRF proteins (41, 42). Therefore, we examined the effects of IC87114 on IRF3 phosphorylation in lungs by western blotting. Three hours following poly I:C administration, IRF3 was phosphorylated in poly I:C-treated mice. Combined treatment with IC87114 and poly I:C significantly increased IRF3 phosphorylation levels compared with mice treated with poly I:C alone (Figure 6). Total IRF3 levels were not affected by poly I:C administration with or without IC87114 treatment (Figure 6).

### A PI3Kδ Inhibitor Suppresses Poly I:C-Induced PD-L1 Expression and Enhances Antiviral IFN Responses in PBECs

**Figure 7:**
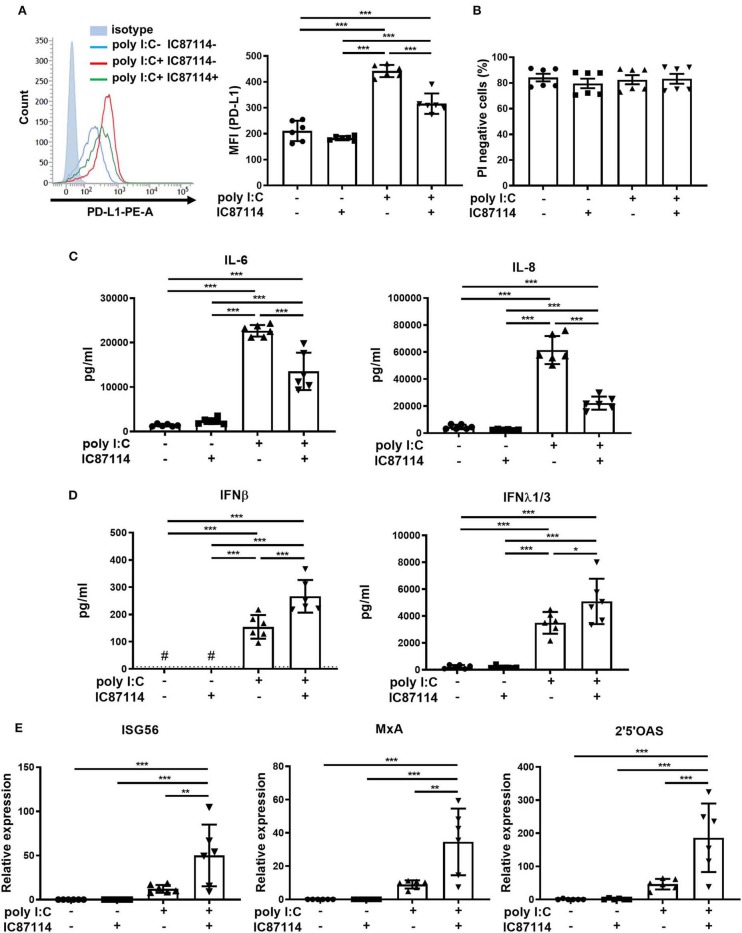
Effects of PI3Kδ inhibitor on poly I:C-induced upregulation of PD-L1, production of cytokines and chemokines, and IFN responses in PBECs. PBECs were pretreated with IC87114 or vehicle for 1 h, then stimulated with poly I:C. Cell lysates for RNA extraction or cells and culture supernatants were collected 6 or 24 h following administration, respectively. **(A,B)** PD-L1 expression **(A)** on PBECs or cell viability **(B)** was analyzed using flow cytometry. Representative histograms are shown. MFI, mean fluorescence intensity. **(C)** Pro-inflammatory cytokine and chemokine levels in supernatants were measured by ELISA. **(D)** IFN levels in supernatants were measured by ELISA. The dotted line shows the lower limit of detection. ^#^Levels in all samples were below the detection limit. **(E)** RNA was extracted and real-time quantitative reverse-transcriptase PCR was performed. Target gene expression levels were normalized to those of 18S rRNA. All results are representative of at least two independent experiments. Data represent means ± SDs (n=6 per group) of three replicates from a minimum of two independent donors. *p < 0.05, **p < 0.01, ***p < 0.001 by one-way ANOVA.

Using bronchial epithelial cells collected from never-smoker patients with normal lung function, we investigated whether IC87114 had similar effects on poly I:C-induced PD-L1 expression, cytokine and chemokine production, and IFN responses as in mouse lung cells. PD-L1 expression on PBECs was significantly increased 24 h following poly I:C stimulation and treatment with IC87114 attenuated poly I:C-induced upregulation of PD-L1 (Figure 7A). At the same time point, no significant difference was observed in cell viability among four groups (Figure 7B and Supplementary Figure 1B). IC87114 also decreased levels of IL-6 and IL-8 induced by poly I:C treatment in culture supernatants 24 h following stimulation (Figure 7C). By contrast, production of IFNβ and λ_1/3_ in culture supernatants was significantly enhanced by combined treatment with IC87114 and poly I:C compared with PBECs treated with poly I:C alone (Figure 7D). We assessed expression of ISG56, MxA and 2′5′OAS 6 h following poly I:C stimulation as representative IRGs in PBECs. Combined treatment with IC87114 and poly I:C significantly increased expression of *ISG56, MxA* and *2*′*5*′*OAS* compared with treatment with poly I:C alone (Figure 7E).

### siRNA Knockdown of the PIK3CD Gene Suppresses Poly I:C-Induced Upregulation of PD-L1 and Enhances Antiviral IFN Responses in PBECs

**Figure 8:**
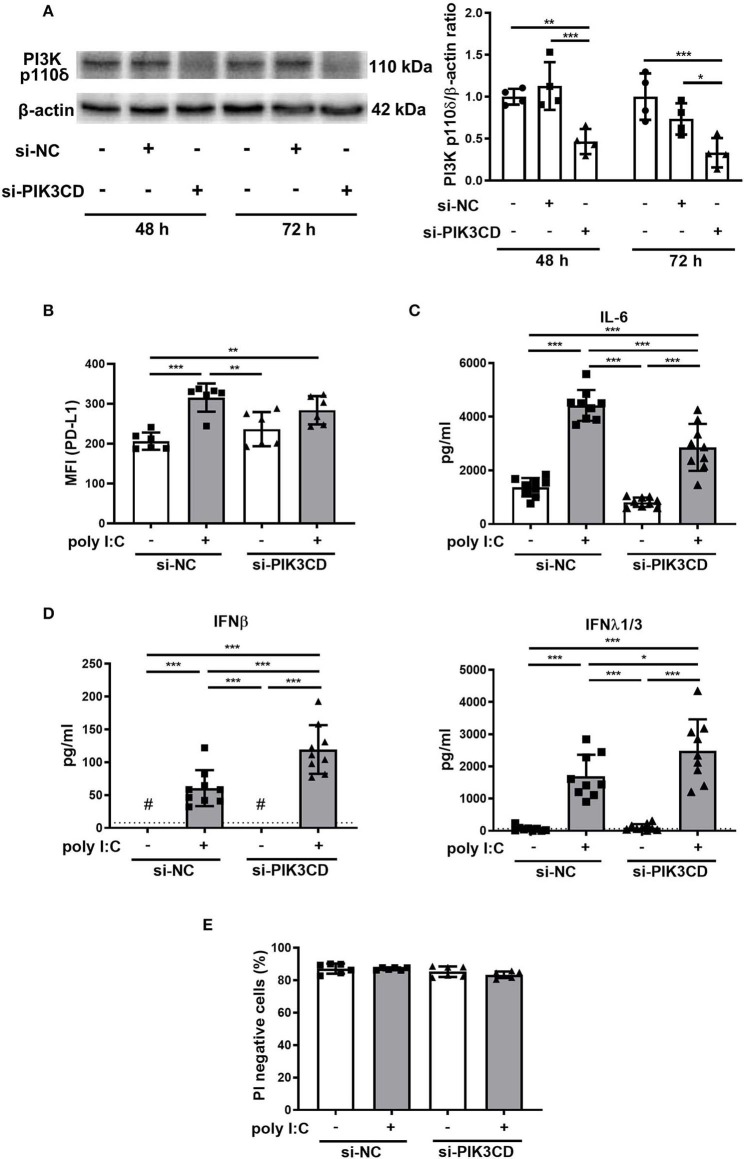
Effects of siRNA targeting the PIK3CD gene on poly I:C-induced upregulation of PD-L1, production of cytokines and IFN responses in PBECs. **(A)** Representative western blots showing PI3K p110δ in PBECs treated with PIK3CD siRNA or negative control (NC) siRNA for 48 or 72 h. Band intensity was quantitated using densitometry. **(B–D)** PBECs were transfected with PIK3CD siRNA or NC siRNA for 48 h, then stimulated with poly I:C or vehicle for 24h. **(B)** PD-L1 expression on PBECs was analyzed using flow cytometry. MFI, mean fluorescence intensity. **(C)** IL-6 levels in supernatants were measured by ELISA. **(D)** IFN levels in supernatants were measured by ELISA. The dotted line shows the lower limit of detection. ^#^Levels in all samples were below the detection limit. **(E)** Viable cells (PI negative) were identified using flow cytometry. All results are representative of at least two independent experiments. Data represent means ± SDs (*n* = 6–9 per group) of three replicates from a minimum of two independent donors. *p < 0.05, **p < 0.01, ***p < 0.001 by one- or two-way ANOVA as appropriate.

The effect of knockdown of the *PIK3CD* gene encoding PI3K p110δ was assessed in PBECs. PI3K p110δ expression was significantly suppressed in PBECs treated with siPIK3CD 48 and 72 h following transfection compared with that in PBECs treated with siNC (Figure 8A). Next, PBECs transfected with siPIK3CD or siNC for 48 h were stimulated with poly I:C and PD-L1 expression and production of cytokine and IFNs in culture supernatants were analyzed 24 h following stimulation. PD-L1 expression was significantly increased on PBECs treated with siNC and poly I:C compared with poly I:C-untreated controls although poly I:C-induced increases in PD-L1 expression were attenuated in PBECs treated with siPIK3CD and poly I:C (Figure 8B). IL-6 production levels in culture supernatants of PBECs treated with siPIK3CD and poly I:C were reduced compared with PBECs treated with siNC and poly I:C (Figure 8C). On the other hand, enhanced production of IFNβ and λ_1/3_ in culture supernatants were observed in PBECs treated with siPIK3CD and poly I:C (Figure 8D). At the same time point, cell viability was assessed and not affected by siRNA knockdown of PIK3CD and stimulation of poly I:C (Figure 8E and Supplementary Figure 1C).

### A PI3Kδ Inhibitor Suppresses hMPV-Induced PD-L1 Expression and Inhibits Viral Replication in PBECs Without Changing the Production Levels of IFNs in Supernatants

**Figure 9:**
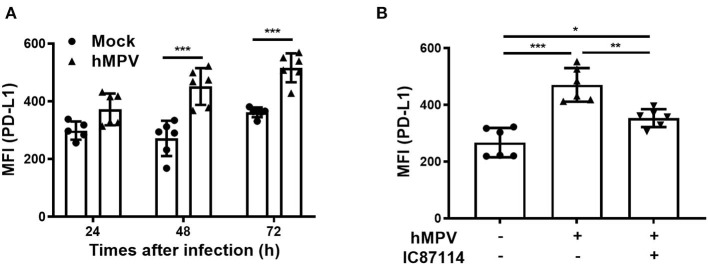
A PI3Kδ inhibitor attenuated hMPV-induced upregulation of PD-L1 on PBECs. **(A)** PBECs were infected with hMPV (MOI 0.1) and PD-L1 expression was analyzed at the indicated times using flow cytometry. **(B)** IC87114 or vehicle was added prior to and after hMPV infection (MOI 0.1). PD-L1 expression was analyzed at 48 hpi using flow cytometry. MFI, mean fluorescence intensity. All results are representative of at least two independent experiments. Data represent means ± SDs (*n* = 6 per group) of three replicates from a minimum of two independent donors. *p < 0.05, **p < 0.01, ***p < 0.001 by one- or two-way ANOVA as appropriate.

**Figure 10:**
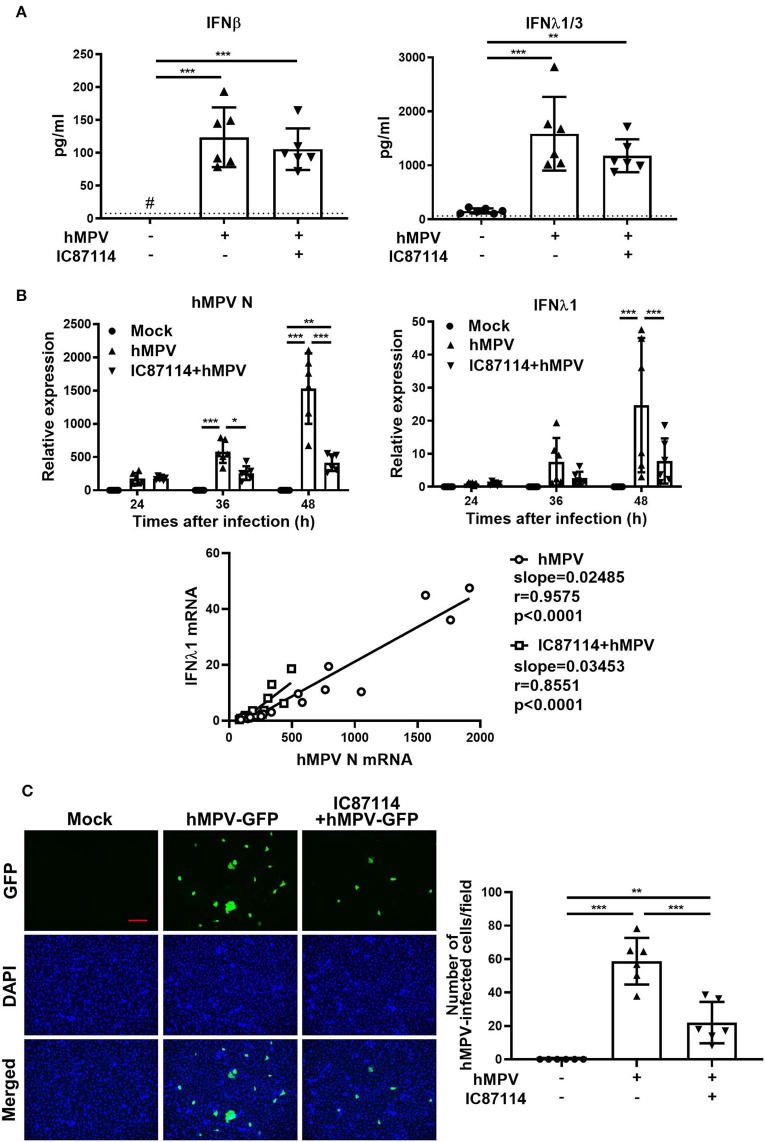
A PI3Kδ inhibitor reduced replication of hMPV in PBECs without changing the production level of IFNs in supernatants. IC87114 or vehicle was added prior to and after hMPV (MOI 0.1) **(A,B)** or hMPV-GFP (MOI 0.1) **(C)** infection. **(A)** Cell culture supernatants were collected at 48 hpi and IFN levels in supernatants were measured by ELISA. The dotted line shows the lower limit of detection. ^#^Levels in all samples were below the detection limit. **(B)** Cell lysates for RNA extraction were collected at 24, 36, and 48 hpi and real-time quantitative reverse-transcriptase PCR was performed. Target gene expression levels were normalized to those of 18S rRNA. **(C)** Images of hMPV-GFP-infected cells at 72 hpi were obtained using fluorescence microscopy (10× objective lens) and the number of cells infected with hMPV-GFP (4× objective lens) was counted. Scale bar, 200 μm. All results are representative of at least two independent experiments. Data represent means ± SDs (*n* = 6 per group) of three replicates from a minimum of two independent donors. *p < 0.05, **p < 0.01, ***p < 0.001 by one- or two-way ANOVA and spearman correlation as appropriate.

Finally, we investigated the effects of IC87114 on hMPV-induced PD-L1 expression, IFN responses and viral replication in PBECs. At 48 and 72 hpi, PD-L1 expression was significantly upregulated on PBECs infected with hMPV at a MOI of 0.1 compared with uninfected controls (Figure 9A). The increases in PD-L1 expression or hMPV nucleocapsid protein (*hMPV N*) and *IFN*λ_*1*_ gene expression were absent if the virus was UV-irradiated thereby excluding non-hMPV induced responses (Supplementary Figures 2A,B). Treatment with IC87114 attenuated hMPV-induced upregulation of PD-L1 at 48 hpi (Figure 9B). Similarly to previous observations (31), hMPV did not induce IL-8 gene in PBECs and protein in culture supernatants (Supplementary Figure 3). IFN production following hMPV infection and viral replication were examined in PBECs treated with or without IC87114. Levels of IFNβ and IFNλ_1/3_ in culture supernatants were significantly increased at 48 hpi and treatment with IC87114 did not affect production levels of IFNβ and IFNλ_1/3_ (Figure 10A). However, *hMPV N* and *IFN*λ_*1*_ gene expression induced by hMPV infection were significantly decreased in PBECs treated with IC87114 at 36 and 48 hpi, respectively (Figure 10B). There was a linear association between *hMPV N* and *IFN*λ_*1*_ gene expression in cells treated with or without IC87114 (Figure 10B). IC87114 changed a slope of a regression line larger, indicating more efficient induction of IFN gene expression responsive to infections in IC87114-treated cells. Next, PBECs were infected with hMPV-GFP at a MOI of 0.1 and observed at 72 hpi by fluorescence microscopy. The number of hMPV-infected cells were significantly decreased and smaller syncytium formation was induced in IC87114-treated cells compared with untreated controls (Figure 10C and Supplementary Figure 4). After infection with UV-irradiated hMPV-GFP, GFP fluorescence and *hMPV N* gene expression was not induced in PBECs (Supplementary Figures 2C,D).

## Discussion

PI3Kδ has been extensively studied as a potential target for anti-oncogenic and anti-inflammatory therapies (27, 43). However, the role of PI3Kδ signaling in antiviral responses is poorly understood. Using a murine model of airway viral infection, we investigated PD-L1 and PD-1 expression on lung cells following i.t. poly I:C administration and observed upregulation of PD-L1 but not PD-1 on epithelial cells, lymphocytes, macrophages, and neutrophils. A selective PI3Kδ inhibitor, IC87114, suppressed poly I:C-induced PD-L1 expression on epithelial cells and neutrophils, but not on lymphocytes and macrophages. This suppressive effects on PD-L1 might be due to inhibition of the Akt/mTOR pathway by IC87114. IC87114 also attenuated poly I:C-induced increases in influx of inflammatory cells and secretion of pro-inflammatory chemokines and cytokines in BALF. Interestingly, IC87114 enhanced poly I:C-induced expression of IFNs and IRGs in the lung and increased the phosphorylation of IRF3. Finally, we assessed the effects of IC87114 in PBECs. Similarly to our *in vivo* results, IC87114 or siRNA knockdown of the *PIK3CD* gene decreased poly I:C-induced PD-L1 expression, production of pro-inflammatory chemokines and cytokines and enhanced antiviral IFN responses in PBECs. Furthermore, IC87114 treatment reduced replication of hMPV in PBECs.

Our *in vivo* experiments showed that the suppressive effects of IC87114 on poly I:C-induced PD-L1 expression depend on cell type. It has been well-established that the upregulation of PD-L1 on infected epithelium causes virus-specific CD8^+^ T cells to fail to eliminate virus in the epithelium (19, 44). Upregulation of PD-L1 on neutrophils was reported to suppress lymphocyte proliferation, induce lymphocyte apoptosis and compromise anti-pathogen defense (45, 46). These previous reports suggested that the suppressive effects of IC87114 on PD-L1 expression by epithelial cells and neutrophils may restore T cell function and promote viral clearance. However, little is known about PD-L1 expression and regulation in macrophages. Loke et al. showed that PD-L1 expression can be induced on macrophages not only by classical activation stimuli such as poly I:C and lipopolysaccharide, but also through alternative activation via IL-4 (47). This alternative induction of PD-L1 may be one reason why IC87114 did not suppress poly I:C-induced PD-L1 expression on macrophages in our study. Additional studies are needed to investigate the functions of PD-L1 on macrophages during immune responses.

Respiratory virus infection precipitates the majority of exacerbations of asthma and COPD. Subsequent production of chemokines and cytokines in the bronchial epithelium induce inflammation in the lungs and excessive inflammatory responses may induce airway hyperresponsiveness and remodeling (48). Previous studies using pan-PI3K inhibitors have showed discrepant results regarding a role for PI3K in TLR-mediated inflammatory responses, suggesting that the effect of PI3K on NF-κB activation may depend on cell type (49, 50). In our study, the selective PI3Kδ inhibitor IC87114 attenuated poly I:C-induced inflammatory responses *in vivo* and *in vitro*. Other investigators also have showed that IC87114 decreased neutrophilic inflammation in BALF to almost control level (51–53). These anti-inflammatory effects of IC87114 might contribute to prevention of asthma and COPD exacerbation. PI3Kδ signaling is thought to play an important role in the pathogenesis of COPD and asthma, both respiratory diseases characterized by chronic lung inflammation (43, 54). In an ovalbumin-induced asthma model, IC87114 treatment or p110δ inactivation in mice decreased type 2 cytokine responses, allergic airway inflammation and airway hyperresponsiveness (55, 56). To et al. reported that PI3Kδ mRNA levels and activity were elevated in the peripheral lung tissues of COPD patients and that IC87114 restored corticosteroid resistance in cigarette smoke-exposed mice through inhibition of oxidative stress-dependent PI3Kδ activation (57). Based on these studies, clinical trials with inhaled PI3Kδ inhibitors in asthmatics and patients with COPD are currently being conducted to evaluate their efficacy in preventing exacerbation of these conditions and maintaining lung function (43, 58).

In this study, we showed that IC87114 significantly enhanced antiviral IFN responses and increased the phosphorylation of IRF3. Phosphorylation of IRF3 is critical for type I interferon production triggered by detection of dsRNA by pathogen recognition receptors (59). Subsequently, binding of secreted IFNs to ubiquitously-expressed type I IFN receptors triggers IFN signaling, followed by induction of IRG expression. A previous study using a pan-PI3K inhibitor showed that the PI3K pathway played an essential role in high concentration of poly I:C-induced phosphorylation of IRF3 (60). However, our results suggest that PI3Kδ may negatively regulate antiviral IFN responses in the lungs. To the best of our knowledge, only two previous studies showed involvement of PI3Kδ in antiviral IFN responses and suppressive effects of a selective PI3Kδ inhibitor on antiviral IFN production. Guiducci et al. reported that IC87114 inhibited IRF7-mediated IFNα production in a dose-dependent manner in human plasmacytoid DCs stimulated by CpG-C, a synthetic TLR9 agonist (61). In another report from Aksoy, IC87114 decreased IFNβ production in LPS-stimulated BMDCs than in IC87114-untreated cells through inhibition of IRF3 phosphorylation (62). PI3Kδ signaling may differentially regulate antiviral IFN responses in different cell types as shown in our previous study in which opposite effects of a pan-PI3K inhibitor on poly I:C-induced PD-L1 expression were observed among bronchial epithelial cells and BMDC (38). As mentioned above, it was reported that PI3Kδ activity was elevated in the peripheral lung tissues of patients with COPD. In addition, a recent study showed that patients with COPD and a history of frequent exacerbations had reduced antiviral IFN responses associated with increased secondary bacterial infections (63). Impaired antiviral IFN responses in asthmatic PBECs were also reported (64, 65). Synthesizing these previous reports and our results, susceptibility to infection may occur though exaggerated PI3Kδ signaling, and PI3Kδ may be targeted therapeutically in COPD and asthma to prevent development and aggravation of viral infection.

Lastly, we demonstrated the effects of IC87114 in PBECs. Bronchial epithelial cells act as the first line of defense against inhaled exogenous substances including viral pathogens recognized by pattern recognition receptors. The expression of PI3Kδ is generally restricted to leukocytes although a few previous studies suggest PI3Kδ expression and activity in non-leukocytes (66–70). Ge et al. showed that PI3Kδ mRNA and protein levels were increased in the airway smooth muscle cells of asthmatics and patients with COPD, respectively (66). In another study, IC87114 decreased hypoxia-inducible factor-1α protein levels in primary airway epithelial cells isolated from OVA-treated mice; this protein stimulates the expression of vascular endothelial growth factor that promotes vascular permeability in respiratory diseases (69). Tumor necrosis factor-α stimulation induced PI3Kδ expression in human endothelial cells and synovial fibroblasts (70). Our results in PBECs are the first demonstration that PI3Kδ signaling may play an important role in upregulation of PD-L1 and negative regulation of IFN responses, resulting in suppression of adaptive and innate responses to viral infections in PBECs. Most importantly, hMPV is able to replicate less efficiently in PBECs treated with IC87114. A limitation of our study was that poly I:C or single-cell experiments are unable to activate adaptive immune responses by presentation of viral antigen. Using live respiratory virus, further *in vivo* investigations are needed to evaluate whether PI3Kδ inhibitors decrease viral loads in lungs and promote recovery from infection.

In summary, a PI3Kδ inhibitor, IC87114, attenuated poly I:C-induced expression of the co-inhibitory molecule PD-L1 on epithelial cells and neutrophils in the lungs. IC87114 also suppressed increases in the influx of inflammatory cells into the lung and secretion of pro-inflammatory chemokines and cytokines. Furthermore, IC87114 enhanced poly I:C-induced antiviral IFN responses in the lungs. Similar results were obtained in *in vitro* experiments using PBECs treated with IC87114 or siRNA knockdown of the *PIK3CD* gene. Finally, experiments using hMPV confirmed that IC87114 inhibits viral replication in PBECs. Taken together, these results indicate that IC87114 may promote virus elimination and clearance, and prevent prolonged lung inflammation exacerbates asthma and COPD. Use of a selective PI3Kδ inhibitor can potentially have significant benefits in virus-exacerbated respiratory diseases.

## Data Availability Statement

The datasets generated for this study are available on request to the corresponding author.

## Ethics Statement

The studies involving human participants were reviewed and approved by the Kyushu University Institutional Review Board for Clinical Research. The patients/participants provided their written informed consent to participate in this study. The animal study was reviewed and approved by the Kyushu University Animal Care and Use Committee.

## Author Contributions

KK, SF, YN, and KM conceived the study design and supervised the scientific work. AF, KK, KT, NY, and TO performed the experiments. AF and KK analyzed the data. All authors contributed to and approved the final manuscript.

### Conflict of Interest

KK received research grants from MSD Life Science Foundation and Public Interest Incorporated Foundation that were unrelated to the submitted work. The remaining authors declare that the research was conducted in the absence of any commercial or financial relationships that could be construed as a potential conflict of interest.

## Supplementary Material

Supplementary Figure 1 Representative dot plots showing a PI profile in mouse lungs or human PBECs. **(A)** IC87114 or vehicle was administered i.t. to mice followed by i.t. administration of poly I:C or vehicle. Viable cells (PI negative) were identified 24 h following administration using flow cytometry. **(B)** PBECs were pretreated with IC87114 or vehicle for 1 h, then stimulated with poly I:C or vehicle. Viable cells (PI negative) were identified 24 h following stimulation using flow cytometry. **(C)** PIK3CD or negative control (NC) siRNA was transfected into PBECs for 48 h, then stimulated with poly I:C or vehicle. Viable cells (PI negative) were identified 24 h following stimulation using flow cytometry.

Supplementary Figure 2 UV-irradiated hMPV was unable to replicate in human bronchial epithelial cells and did not induce PD-L1 expression and IFN responses. **(A,B)** PBECs **(A)** or BEAS-2B **(B)** were infected with hMPV (MOI 0.1) or UV-irradiated hMPV (MOI 0.1). **(A)** PD-L1 expression was analyzed at 24 hpi using flow cytometry. **(B)** Cell lysates for RNA extraction were collected at 48 and 72 hpi and real-time quantitative reverse-transcriptase PCR was performed. **(C,D)** PBECs were infected with hMPV-GFP (MOI 0.1) or UV-irradiated hMPV-GFP (MOI 0.1). **(C)** Images of infected cells at 72 hpi obtained using fluorescence microscopy. Scale bar, 100 μm. **(D)** Cell lysates for RNA extraction were collected at 120 hpi and real-time quantitative reverse-transcriptase PCR was performed. Target gene expression levels were normalized to those of 18S rRNA. Data represent means ± SDs (*n* = 3 per group). ^*^*p* < 0.05, ^**^*p* < 0.01 by one- or two-way ANOVA as appropriate.

Supplementary Figure 3 hMPV did not induce IL-8 gene expression in PBECs and protein expression in supernatants. IC87114 or vehicle was added prior to and after hMPV (MOI 0.1) infection. **(A)** Cell lysates for RNA extraction were collected at 24, 36, and 48 hpi and real-time quantitative reverse-transcriptase PCR was performed. Target gene expression levels were normalized to those of 18S rRNA. **(B)** Cell culture supernatants were collected at 24 and 48 hpi and IL-8 levels in supernatants were measured by ELISA. All results are representative of at least two independent experiments. Data represent means ± SDs (*n* = 6 per group) of three replicates from a minimum of two independent donors. ^*^*p* < 0.01 by two-way ANOVA.

Supplementary Figure 4 Images of hMPV-GFP-infected cells. PBECs were cultured to semi-confluence in 12-well plates. IC87114 or vehicle was added prior to and after hMPV-GFP (MOI 0.1) infection, then cells were obsetved at 72 hpi using fluorescence microscopy.

Supplementary Table 1 Sequences of real-time PCR primers used in this study.
