# Supplementary figures and images for: Inhibition of PI3Kδ Enhances Poly I:C-Induced Antiviral Responses and Inhibits Replication of Human Metapneumovirus in Murine Lungs and Human Bronchial Epithelial Cells
Source: Front Immunol. 2020 Mar 11;11:432. doi: 10.3389/fimmu.2020.00432 (PMC7079687; doi:10.3389/fimmu.2020.00432)

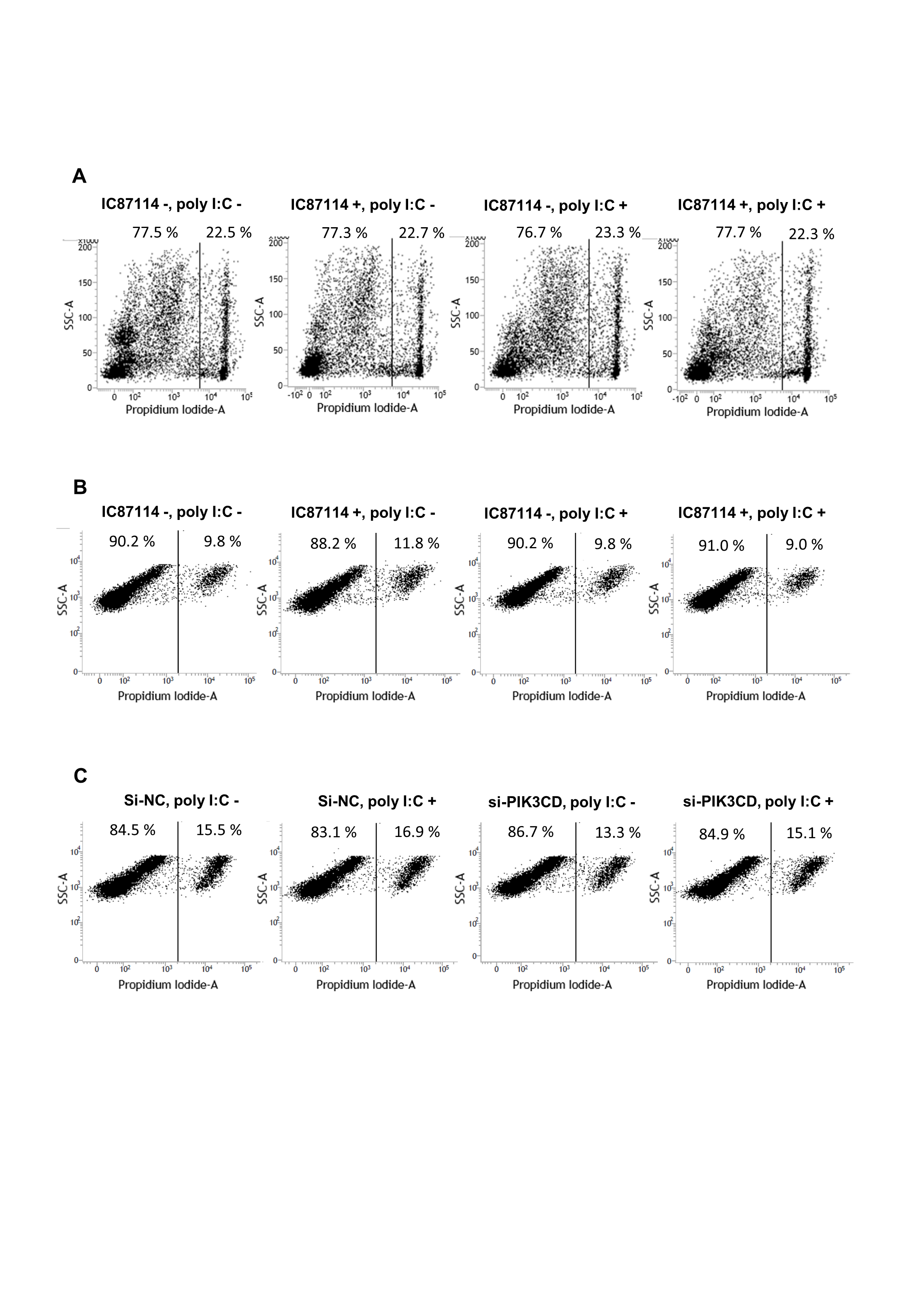

Supplement: Supplementary Figure 1 — Representative dot plots showing a PI profile in mouse lungs or human PBECs. (A) IC87114 or vehicle was administered i.t. to mice followed by i.t. administration of poly I:C or vehicle. Viable cells (PI negative) were identified 24 h following administration using flow cytometry. (B) PBECs were pretreated with IC87114 or vehicle for 1 h, then stimulated with poly I:C or vehicle. Viable cells (PI negative) were identified 24 h following stimulation using flow cytometry. (C) PIK3CD or negative control (NC) siRNA was transfected into PBECs for 48 h, then stimulated with poly I:C or vehicle. Viable cells (PI negative) were identified 24 h following stimulation using flow cytometry. [file Image_1.TIF]

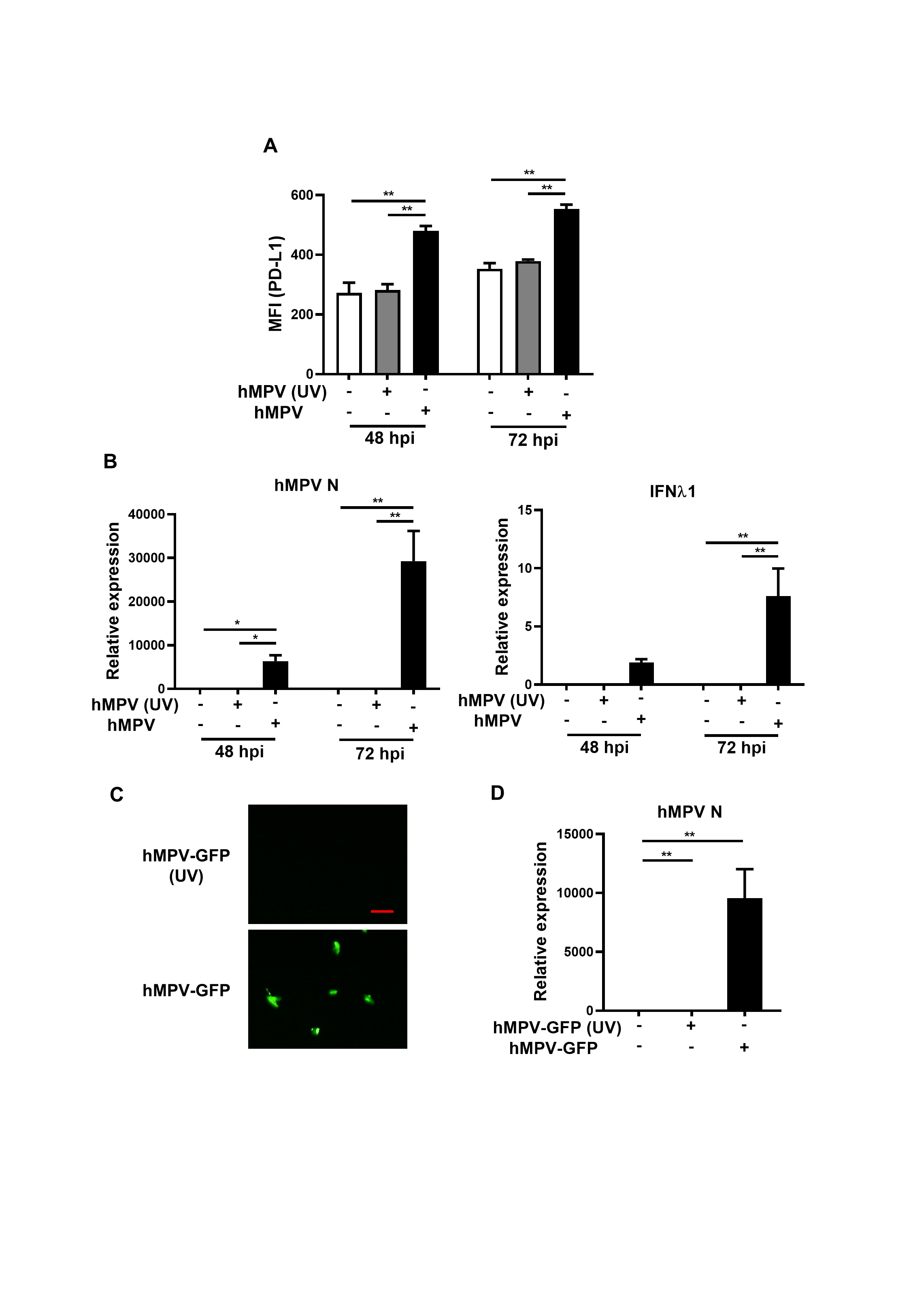

Supplement: Supplementary Figure 2 — UV-irradiated hMPV was unable to replicate in human bronchial epithelial cells and did not induce PD-L1 expression and IFN responses. (A,B) PBECs (A) or BEAS-2B (B) were infected with hMPV (MOI 0.1) or UV-irradiated hMPV (MOI 0.1). (A) PD-L1 expression was analyzed at 24 hpi using flow cytometry. (B) Cell lysates for RNA extraction were collected at 48 and 72 hpi and real-time quantitative reverse-transcriptase PCR was performed. (C,D) PBECs were infected with hMPV-GFP (MOI 0.1) or UV-irradiated hMPV-GFP (MOI 0.1). (C) Images of infected cells at 72 hpi obtained using fluorescence microscopy. Scale bar, 100 μm. (D) Cell lysates for RNA extraction were collected at 120 hpi and real-time quantitative reverse-transcriptase PCR was performed. Target gene expression levels were normalized to those of 18S rRNA. Data represent means ± SDs (n = 3 per group). *p < 0.05, **p < 0.01 by one- or two-way ANOVA as appropriate. [file Image_2.TIF]

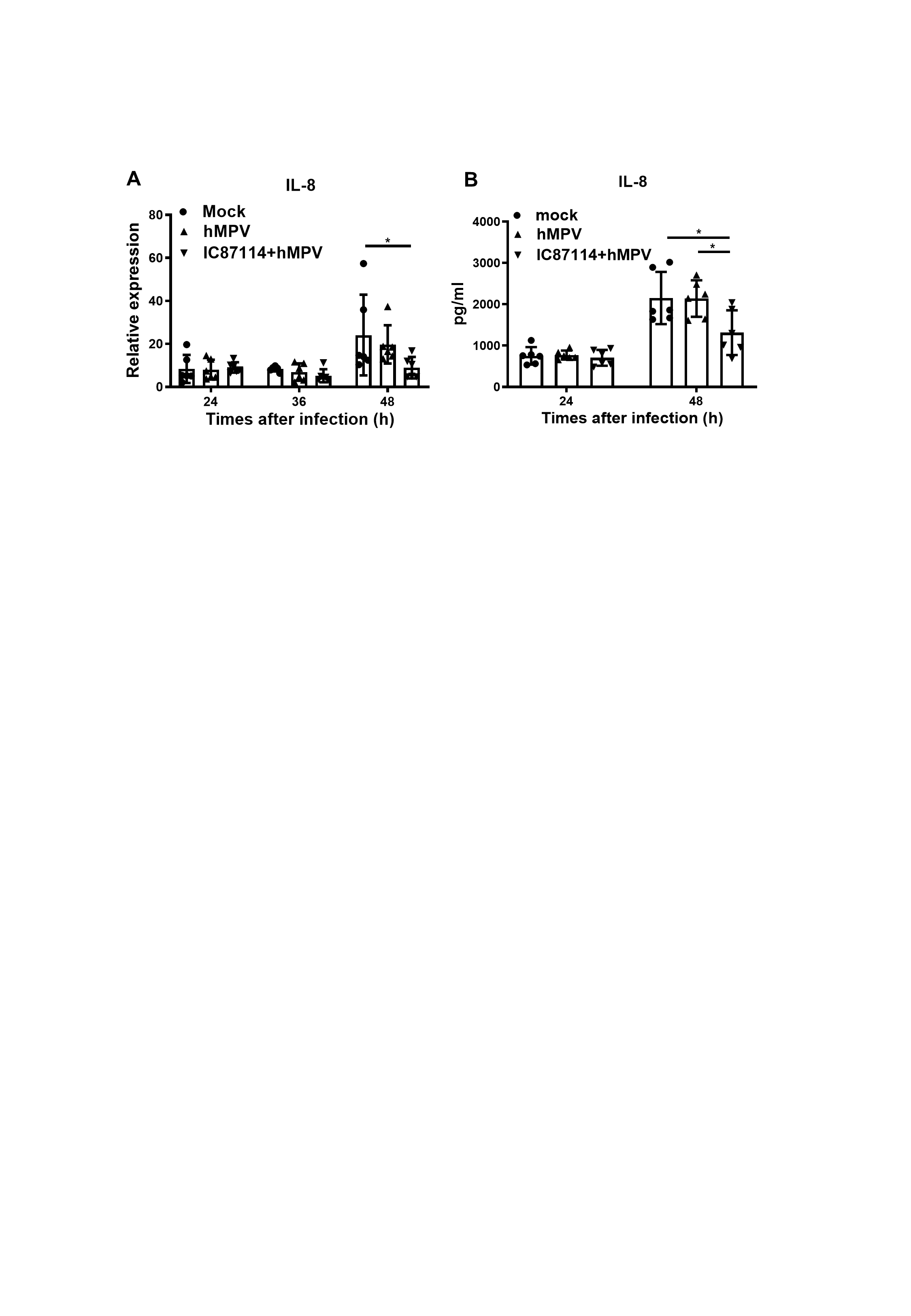

Supplement: Supplementary Figure 3 — hMPV did not induce IL-8 gene expression in PBECs and protein expression in supernatants. IC87114 or vehicle was added prior to and after hMPV (MOI 0.1) infection. (A) Cell lysates for RNA extraction were collected at 24, 36, and 48 hpi and real-time quantitative reverse-transcriptase PCR was performed. Target gene expression levels were normalized to those of 18S rRNA. (B) Cell culture supernatants were collected at 24 and 48 hpi and IL-8 levels in supernatants were measured by ELISA. All results are representative of at least two independent experiments. Data represent means ± SDs (n = 6 per group) of three replicates from a minimum of two independent donors. *p < 0.01 by two-way ANOVA. [file Image_3.TIF]

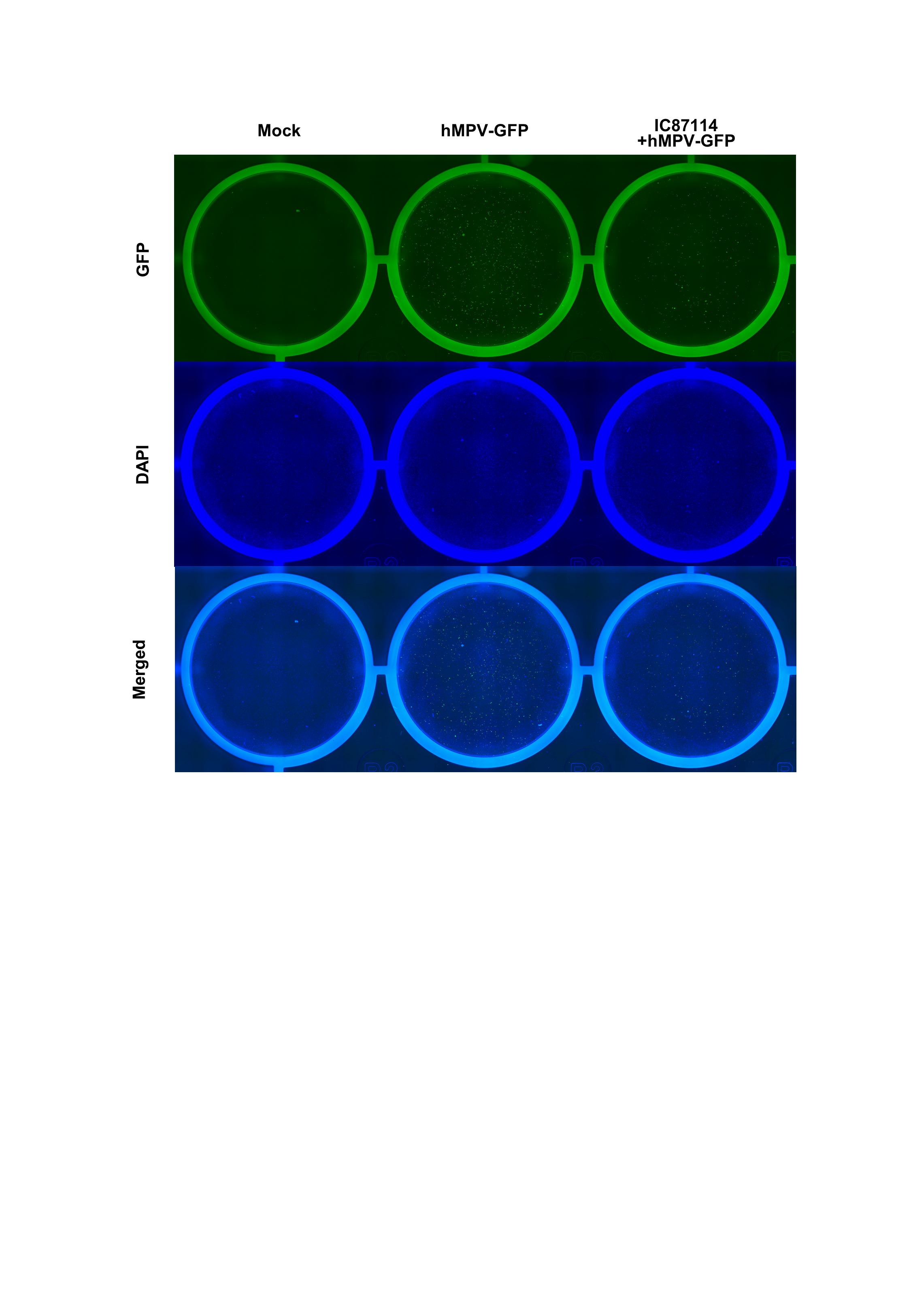

Supplement: Supplementary Figure 4 — Images of hMPV-GFP-infected cells. PBECs were cultured to semi-confluence in 12-well plates. IC87114 or vehicle was added prior to and after hMPV-GFP (MOI 0.1) infection, then cells were obsetved at 72 hpi using fluorescence microscopy. [file Image_4.TIF]
