# Supplementary material for: Inhibition of PI3Kδ Enhances Poly I:C-Induced Antiviral Responses and Inhibits Replication of Human Metapneumovirus in Murine Lungs and Human Bronchial Epithelial Cells
Source: Front Immunol. 2020 Mar 11;11:432. doi: 10.3389/fimmu.2020.00432 (PMC7079687; doi:10.3389/fimmu.2020.00432)
Supplement: Supplementary Table 1 — Sequences of real-time PCR primers used in this study. [file Table_1.DOC]

**Supplementary Table 1**

Sequences of real-time PCR primers used in this study.

*Mouse Gapdh*  Sense: 5’ CAT GGC CTT CCG TGT TCC TA 3’

Antisense: 5’ GCG GCA CGT CAG ATC CA 3’

*Mouse Ifn*β Sense: 5’ CCCTATGGAGATGACGGAGA 3’

Antisense: 5’ ACCCAGTGCTCTGGAGAAATTG 3’

*Mouse Ifn*λ_2_ Sense: 5’ CAGACCTGTACACAGCTTCAGG 3’

Antisense: 5’ GGGTGAGCAGGCGAAACA 3’

*Mouse Ifit1* Sense: 5’ TCAAGGCAGGTTTCTGAGGA 3’

Antisense: 5’ ACCTGGTCACCATCAGCATT 3’

*Mouse Ifit2* Sense: 5’ AAGGCAGAGGAAGAGGTTGC 3’

Antisense: 5’ GTCGCAGATTGCTCTCCAGT 3’

*Mouse Isg15* Sense: 5’ TGAGAGCAAGCAAGCCAGAAG 3’

Antisense: 5’ ACGGACACCAGGAAATCGTT 3’

*Human 18S* Sense: 5’ AAA CGG CTA CCA CAT CCA AG 3’

Antisense: 5’ CCT CCA ATG GAT CCT CGT TA 3’

*Human PD-L1* Sense: 5’ CAATGTGACCAGCACACTGAGAA 3’

Antisense: 5’ GGCATAATAAGATGGCTCCCAGAA 3’

*Human Isg56* Sense: 5’ GCA GCC AAG TTT TAC CGA AG 3’

Antisense: 5’ CAC CTC AAA TGT GGG CTT TT 3’

*Human Mxa* Sense: 5’ GTG CAT TGC AGA AGG TCA GA 3’

Antisense: 5’ TTC AGG AGC CAG CTG TAG GT 3’

*Human 2’5’Oas* Sense: 5’ ACA GGC AGA AGA GGA CTG GA 3’

Antisense: 5’ TAG AAG GCC AGG AGT CAG GA 3’

*Human IFNλ* _1_  Sense: 5’ CTAGACCAGCCCCTTCACAC 3’

Antisense: 5’ AAGGTGACAGATGCCTCCAG 3’

*Human IL-8* Sense: 5’ TCTGCAGCTCTGTGTGAAGG 3’

Antisense: 5’ AACCCTCTGCACCCAGTTTTCCTTG 3’

*hMPV N protein* Sense: 5’ CAGAGAGAGTACAGCAGATTCTAA 3’

Antisense: 5’ TTCTCTACTCCGTGTATGTCTAAC 3’
